# Prescribing Trends for Bipolar Disorder Drugs in Alberta, Canada Between 2008 and 2021: Tendances en matière de prescription de médicaments pour le trouble bipolaire en Alberta, au Canada, entre 2008 et 2021

**DOI:** 10.1177/07067437251355643

**Published:** 2025-07-10

**Authors:** Samreen Shafiq, Paul Everett Ronksley, Meghan Jessica Elliott, Andrew Gabriel McKay Bulloch, Scott Burton Patten

**Affiliations:** 1 Department of Community Health Sciences, 2129 University of Calgary, Calgary, Alberta, Canada; 2 Department of Psychiatry, 2129 University of Calgary, Calgary, Alberta, Canada

**Keywords:** lithium, second-generation antipsychotics, antidepressants, anticonvulsants, bipolar disorder, prevalence, incidence, drug utilization, pharmacoepidemiology

## Abstract

**Aims:**

The approval of new drugs for bipolar disorder (BD) may have caused a shift in prescribing trends among patients with BD. The objective of the study was to describe prescribing trends amongst individuals with BD in Alberta, Canada.

**Methods:**

This study used provincial administrative health data from Alberta, Canada. Individuals with at least one ICD-9 or ICD-10 code for BD were identified from three databases – Provider claims, Hospital Discharge Abstract Database and the Ambulatory Care Classification System. Within this cohort, we identified prevalent, new and combination use of commonly prescribed BD drugs through prescription information from the Pharmaceutical Information Network database.

**Results:**

Between April 1, 1994, and March 31, 2021, 136,628 individuals had at least 1 code of BD with 9,466,407 prescriptions dispensed between January 1, 2008 to March 31, 2021. New users of all drugs declined over time, especially from 2019 to 2021. Among all BD drugs, antidepressants were the most commonly prescribed in both prevalent and new users throughout the study period. Among recommended treatments for BD, quetiapine was one of the most prescribed drugs amongst prevalent users. An overall decline was noted in prescribing of lithium, divalproex and carbamazepine among prevalent and new users. Most individuals were prescribed a single drug for BD treatment. The most common combination therapy for prevalent users was an antidepressant with a second-generation antipsychotic (SGA).

**Conclusions:**

Overall, we uncovered a concerning trend in the prescribing patterns for BD treatment, with antidepressants and SGAs being prescribed frequently and a decline in prescribing of lithium and other mood stabilizers. This study emphasizes the need for initiatives promoting evidence-based guidelines and better alignment with best practices for managing BD in outpatient settings.

**Plain Language Summary Title:**

Prescribing Trends for Bipolar Disorder Drugs in Alberta, Canada Between 2008 to 2021

## Plain Language Summary

This study examined prescribing trends for bipolar disorder (BD) in Alberta, Canada, using provincial health data. Among over 136,000 individuals with BD, prescription data from 2008 to 2021 showed a decline in new users of all BD medications, especially after 2019. Antidepressants were the most commonly prescribed drugs, followed by second-generation antipsychotics (SGAs) like quetiapine. In contrast, the use of mood stabilizers such as lithium, divalproex, and carbamazepine declined. Most patients received monotherapy, with the most common combination being an antidepressant plus an SGA. These trends highlight a shift away from traditional mood stabilizers and underscore the need for improved alignment of clinical practice and evidence-based BD treatment guidelines.

## Résumé

**Objectifs:**

L’approbation de nouveaux médicaments pour le trouble bipolaire pourrait avoir entraîné un changement dans les tendances de prescription chez les patients atteints de trouble bipolaire. L’étude visait à décrire les tendances en matière de prescription chez les personnes atteintes de trouble bipolaire en Alberta, au Canada.

**Méthodes:**

Cette étude a utilisé les données administratives provinciales sur la santé de l’Alberta, au Canada. Les personnes présentant au moins un code CIM-9 ou CIM-10 de trouble bipolaire ont été identifiées à partir de trois bases de données : les demandes de remboursement des prestataires, la base de données de sortie d’hôpital et le fichier du système de classification des soins ambulatoires. Au sein de cette cohorte, nous avons identifié l'utilisation actuelle, nouvelle et combinée des médicaments couramment prescrits pour le trouble bipolaire à partir des renseignements figurant sur les prescriptions provenant de la base de données du Pharmaceutical Information Network.

**Résultats:**

Entre le 1er avril 1994 et le 31 mars 2021, 136 628 personnes ont présenté au moins un code de trouble bipolaire et 9 466 407 ordonnances ont été délivrées entre le 1er janvier 2008 et le 31 mars 2021. Le nombre de nouveaux utilisateurs de tous les médicaments a diminué au fil du temps, en particulier entre 2019 et 2021. Parmi tous les médicaments prescrits pour le trouble bipolaire, les antidépresseurs ont été les plus couramment prescrits, tant chez les utilisateurs actuels que chez les nouveaux utilisateurs, tout au long de la période étudiée. Parmi les traitements recommandés pour le trouble bipolaire, la quétiapine était l’un des médicaments les plus prescrits chez les utilisateurs actuels. On a observé une diminution générale de la prescription de lithium, de divalproex et de carbamazépine chez les utilisateurs actuels et les nouveaux utilisateurs. La plupart des personnes se sont vu prescrire un seul médicament pour le traitement du trouble bipolaire. Le traitement combiné le plus courant chez les utilisateurs actuels était un antidépresseur avec un antipsychotique de deuxième génération (SGA).

**Conclusions:**

Dans l’ensemble, nous avons découvert une tendance préoccupante dans les habitudes de prescription pour le traitement du trouble bipolaire, avec une prescription fréquente d’antidépresseurs et de SGA, et une baisse de la prescription de lithium et d’autres stabilisateurs de l’humeur. Cette étude souligne la nécessité de mettre en place des initiatives visant à promouvoir des lignes directrices fondées sur des données probantes et alignées sur des pratiques optimales pour la prise en charge du trouble bipolaire en milieu ambulatoire.

## Introduction

Recent advancements in bipolar disorder (BD) treatment offer patients a large range of management and treatment options. Along with older drugs such as lithium and divalproex, second-generation antipsychotics (SGA) are currently recommended treatments for both acute and maintenance treatment of manic and depressive episodes.^1,2^ Simultaneously, cautious use of antidepressants is permitted for treatment of acute depressive episodes in bipolar I disorder (BD I) according to recommendations. However, antidepressants are generally reserved for select cases with mood stabilizers due to occurrence of mood destabilization and rapid cycling caused by antidepressant monotherapy.^2–4^

Despite evidence of similar prescribing determinants, newer drugs may have led to a shift in prescribing practices worldwide. Prior drug utilization studies revealed a decline in the use of older options in favour of these newer therapies. In the US, from 1996 to 2016, antipsychotic prescriptions for BD patients in outpatient settings rose five-fold, while the use of other mood stabilizers dropped from 62.3% to 26.4%.^5^ Antidepressant prescriptions remained steady, with around 50% of patients consistently receiving them throughout the study period. Similarly, in Scotland between 2009 and 2016, antipsychotic use increased from 45.8% to 51.1%, while antidepressant use remained stable at around 60%.^6^ Although lithium prescriptions decreased, the use of anti-epileptics, particularly valproate, increased amongst men.

Although there are several monotherapies recommended for acute and maintenance treatment of BD episodes, combination therapies may be required in treatment-resistant cases. A systematic review by Fornaro et al. found that 85% of BD patients use two or more drugs, and 36% patients use four or more drugs concurrently, potentially increasing the risk of side effects and drug interactions.^7^ On the other hand, a study of psychiatric practices in Germany reported that 60% of patients were on monotherapy in 2009, where this decreased by 2% in 2018, whereas, 26.1% were on combination therapy in 2009 and increased to 27.7% in 2018.^8^

A Canadian study (2002–2010) showed a significant rise in prescribers’ preference for atypical antipsychotics for BD, despite comprehensive clinical guidelines offering various treatment options.^9^ This raises concerns about a potential gap between recommended practices and real-world drug use. Building on our previous research on lithium usage in Alberta (which showed relative stability in use), this study explores current trends in drug use for BD in the province.^10^ Our objective was to describe the prescribing trends of drugs used in BD amongst individuals with International Classification of Diseases, Ninth revision (ICD-9) or Tenth revision (ICD-10) code for BD (prevalent use). We also examined new use of these medications and use in combination therapies between 2008 and 2021.

## Methods

### Data Source and Study Population

Administrative health data linking pharmacy dispensation data with health encounters for all adults (≥18 years) was obtained from the Ministry of Health within Alberta, Canada. The datasets were linked through a unique patient identifier. Individuals with at least one BD ICD-9 or ICD-10 code were identified from provider claims (from April 1, 1994, to March 31, 2021), Hospital Discharge Abstract Database (April 1, 2002, to March 31, 2021), and the Ambulatory Care Classification System file (from April 1, 2002, to March 31, 2021). The provider claims include outpatient visits to physician and uses ICD-9 codes for diagnosis of conditions. The Hospital Discharge Abstract Database and Ambulatory Care Classification System file contains the hospital admissions and emergency care visits, respectively. Over the time period studied, these databases used ICD-10 codes for diagnoses of conditions. We used ICD-9 codes 296.0–296.1, 296.4–296.9 and ICD-10 codes F31 to identify individuals with probable BD. These data sources were also linked to the provincial health insurance registry, which provides information on patient sex, date of birth, migration or death status.

For this cohort, we identified prescribed medications for BD within the provincial Pharmaceutical Information Network (PIN) database from January 1, 2008 to March 31, 2021. The PIN contains dispensing information (i.e. date of dispensing, quantity, duration, drug form, drug identification number and prescriber information). We specifically identified prescriptions of aripiprazole, asenapine, carbamazepine, divalproex, lamotrigine, lithium, lurasidone, olanzapine, quetiapine and risperidone, bupropion, selective serotonin reuptake inhibitors (SSRI), tricyclic antidepressants (TCA) and other antidepressants (including serotonin and norepinephrine reuptake inhibitors (SNRI), serotonin modulators, and noradrenergic and specific serotonergic antidepressants). We examined certain drugs within the categories of SGAs (aripiprazole, asenapine, lurasidone, olanzapine, quetiapine, risperidone), mood stabilizers (carbamazepine, divalproex, lamotrigine, lithium) and bupropion separately, as these drugs are recommended hierarchically within Canadian Network for Mood and Anxiety Treatments (CANMAT) for BD.^2^ All prescription records prior to first BD claim dates were excluded to reduce the risk of misclassifying medications used for earlier major depressive disorder (MDD) episodes. Ethical approval was obtained from the University of Calgary's Conjoint Bioethics Review Board, which granted waiver of patient consent.

### Study Outcomes

#### Prevalent and New Users

The prevalent and new users of BD drugs were reported by quarterly time blocks. For prevalent use, we identified patients with at least one BD drug prescription within a given quarter. This quarterly prevalence was reported from January 1, 2008, until March 31, 2021. For new users, the first quarter of use of every BD drug was identified as those with a first prescription and no record of a prescription for the same drug in the prior year. We report estimates from 2009 onward to allow for a consistent 1-year look back window for all study participants. The proportion of prevalent and new users were determined amongst the entire cohort of individuals with a BD code (denominator) identified from this study. This denominator changed over the study timeframe, due to the emergence of new patients with BD codes, deaths and migration occurring over time. The treatment rates of prevalent and new users were reported per 1,000 persons overall. The population size in each quarter was taken as an approximation of person-time within the quarter, providing a denominator for the quarterly rates. For prevalent users, we examined total use and the use of both monotherapy and combination therapies.

#### Monotherapy and Combination Use

To assess combination therapies, we examined the number of prescription drugs each patient in the BD cohort received per quarter. This allowed us to categorize patients into monotherapy (using one of the investigated drugs) or combination therapy (using two or more of the investigated drugs). This report details the utilization of the following drugs for monotherapy and combination therapy: carbamazepine, divalproex, lamotrigine, lithium, SGAs and antidepressants.

### Statistical Analysis

To provide a smoothed description of the trends, quarterly changes in new and prevalent drug use among adults with BD were initially examined using linear regression. However, linearity assumptions were found to be violated and therefore alternate modelling strategies were explored guided by principles of parsimony assessed using the deviance difference test, Akaike Information Criteria and Bayesian Information Criteria for each model.^11^ In addition, each model was plotted against a scatter plot with 95% confidence intervals (CIs) to help ensure that temporal trends were captured.

To assess the potential influence of coding errors on the prevalence of antidepressant use in individuals with BD, we performed a sensitivity analysis focusing on those with two or more BD ICD codes. We compared the proportion of antidepressant users among individuals with two BD codes to those with one code. This approach aimed to explore the potential impact of misclassification, specifically a lack of specificity that might arise from the use of a single code.

## Results

### Cohort Characteristics

Between April 1, 1994, and March 31, 2021, 136,628 individuals had at least 1 code of BD with 15,043,460 prescriptions dispensed between January 1, 2008, and March 31, 2021. During the study period, 42.8% (95% CI, 42.5 to 43.0%) of patients were male and 57.2% (95% CI, 57.0 to 57.5%) were female. Within this cohort, 12.7% (95% CI, 12.5 to 12.9%) were 18–24 years at the time of treatment initiation (for those with a record of drug prescriptions within this study timeframe) or first recorded date of diagnosis (for those not on one of the investigated drugs), 50.0% (95% CI, 49.8 to 50.3%) were 25–49 years, 21.7% (95% CI, 21.5 to 21.9%) were 50–64 years and 15.6% (95% CI, 15.4 to 15.8%) were 65 and over. Throughout the study quarters, the percentage of individuals without a prescription for any of the investigated BD drugs ranged from 5% to 14%.

### Prevalent Users

**Figure 1.**
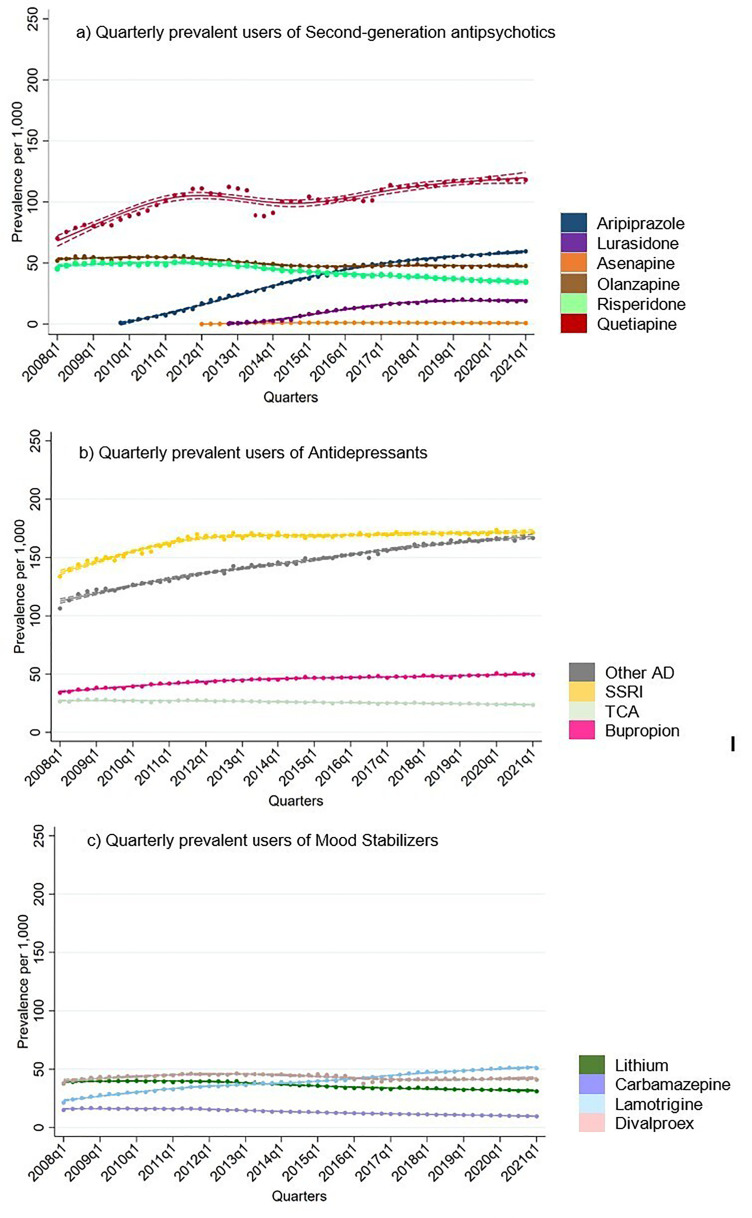
Quarterly prevalent users of second-generation antipsychotics, antidepressants and mood stabilizers in total population.

Among all BD drugs, SSRIs were most commonly prescribed throughout the study period by a large margin (Figure 1(b)). In the beginning of 2008, treatment rate for SSRIs was 134 per 1,000, which steadily increased to and remained constant at 169 per 1,000 by the 4^th^ quarter of 2011. Treatment rates of other antidepressants showed a continuous increase from 106 to 166 per 1,000 observed over the study period.

**Figure 2.**
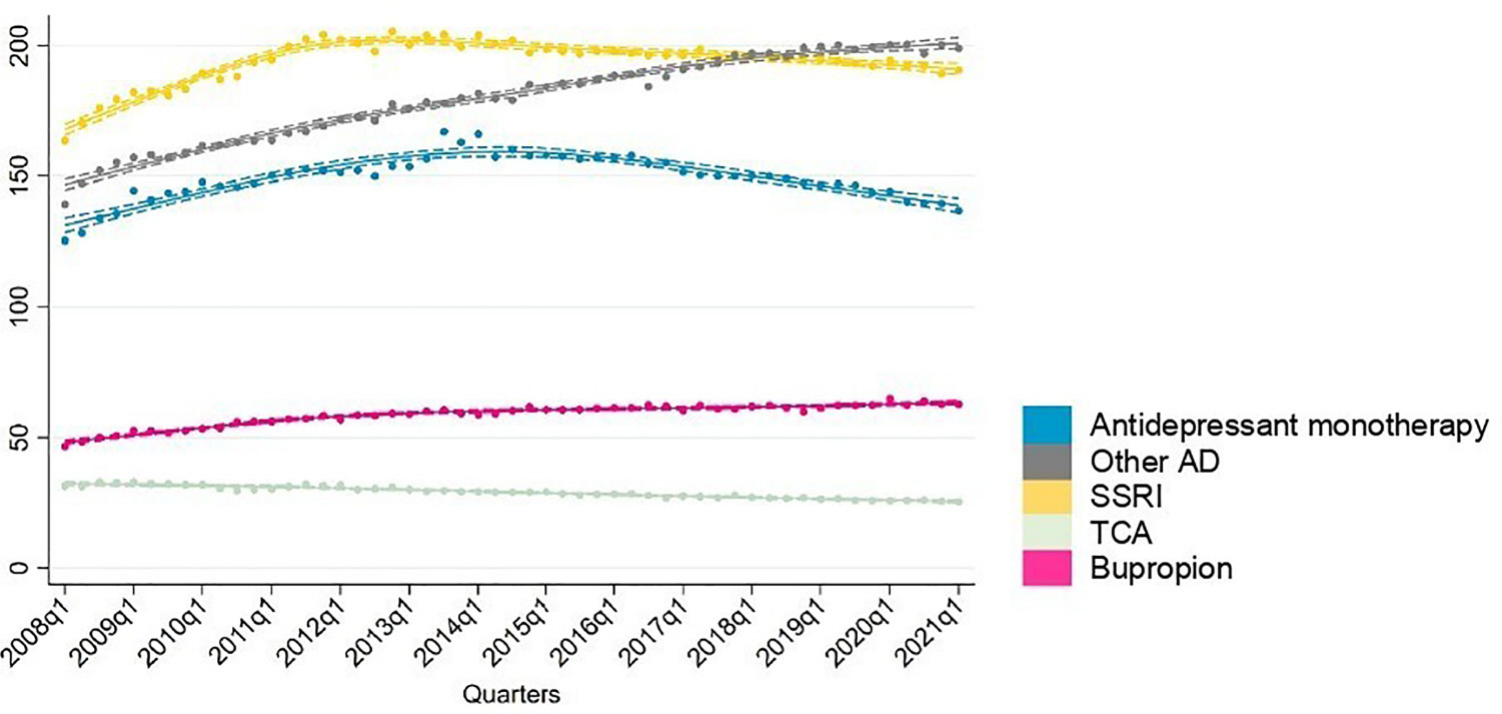
Sensitivity analysis of prevalent antidepressant users in those with two or more codes.

Sensitivity analyses indicated similar trends for individuals with one or multiple BD codes (Figure 2). Amongst BD patients with multiple codes, treatment rates of SSRIs increased from 164 per 1,000 to 190 per 1,000 by 2021. Similarly, other antidepressant and bupropion treatment rates increased steadily throughout 2008 to 2021.

Treatment rates of carbamazepine were the lowest among the BD drugs studied, with a downward trend throughout the observation period (Figure 1(c)). Treatment rates of lithium and divalproex were similar at the beginning of the study period (around 38 per 1,000 in the first quarter of 2008). However, treatment rates of lithium decreased to 31 per 1,000 by 2021, while, treatment rates of divalproex increased to 45 per 1,000 by 2011 and then decreased to 41 by 2021. Only lamotrigine treatment rates appeared to increase throughout the study period from 31 to 51 per 1,000.

Amongst the SGAs, treatment rates for quetiapine were the highest throughout the study period, which started at 70 per 1,000 in 2008 and increased to 111 per 1,000 by 2012. A decrease in quetiapine treatment rates was observed between 2013 and 2016, followed by an increase to 118 per 1,000 by 2021. Aripiprazole and lurasidone were introduced into the market during the study period (between 2008 and 2021). Treatment rates of aripiprazole showed a steady rise, reaching 60 per 1,000 by 2021. Olanzapine and risperidone treatment rates declined throughout the observation period.

### New Users

**Figure 3.**
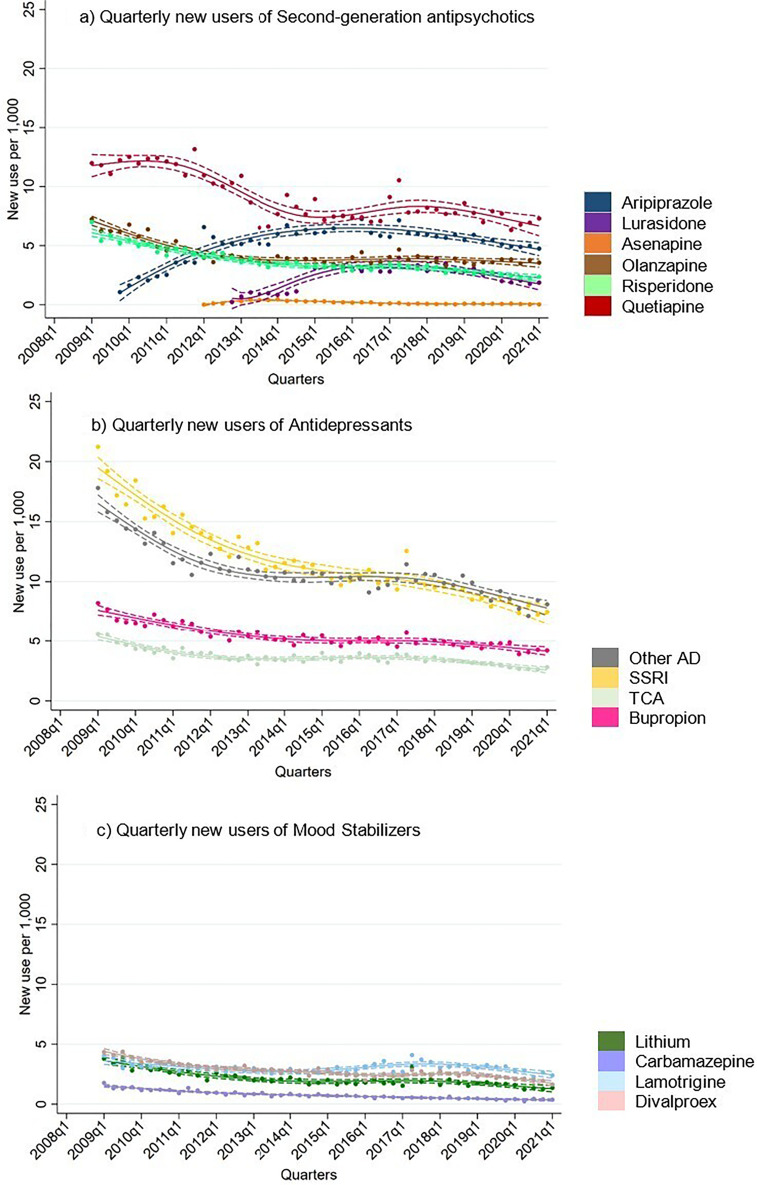
Quarterly new users of second-generation antipsychotics, antidepressants and mood stabilizers in total population.

Overall, new users for all drug classes appeared to decrease over time (Figure 3). Antidepressants had the highest treatment rates of new users throughout the study period. Treatment rates of new users for all mood stabilizers initially decreased and then remained stable until the end of 2019. In contrast, new users of aripiprazole and lurasidone, both SGAs introduced during the study period, increased steadily until 2019. Interestingly, data from 2020 to 2021 suggests a potential decrease in new users for all drug classes.

### Monotherapy and Combination Therapy

**Figure 4.**
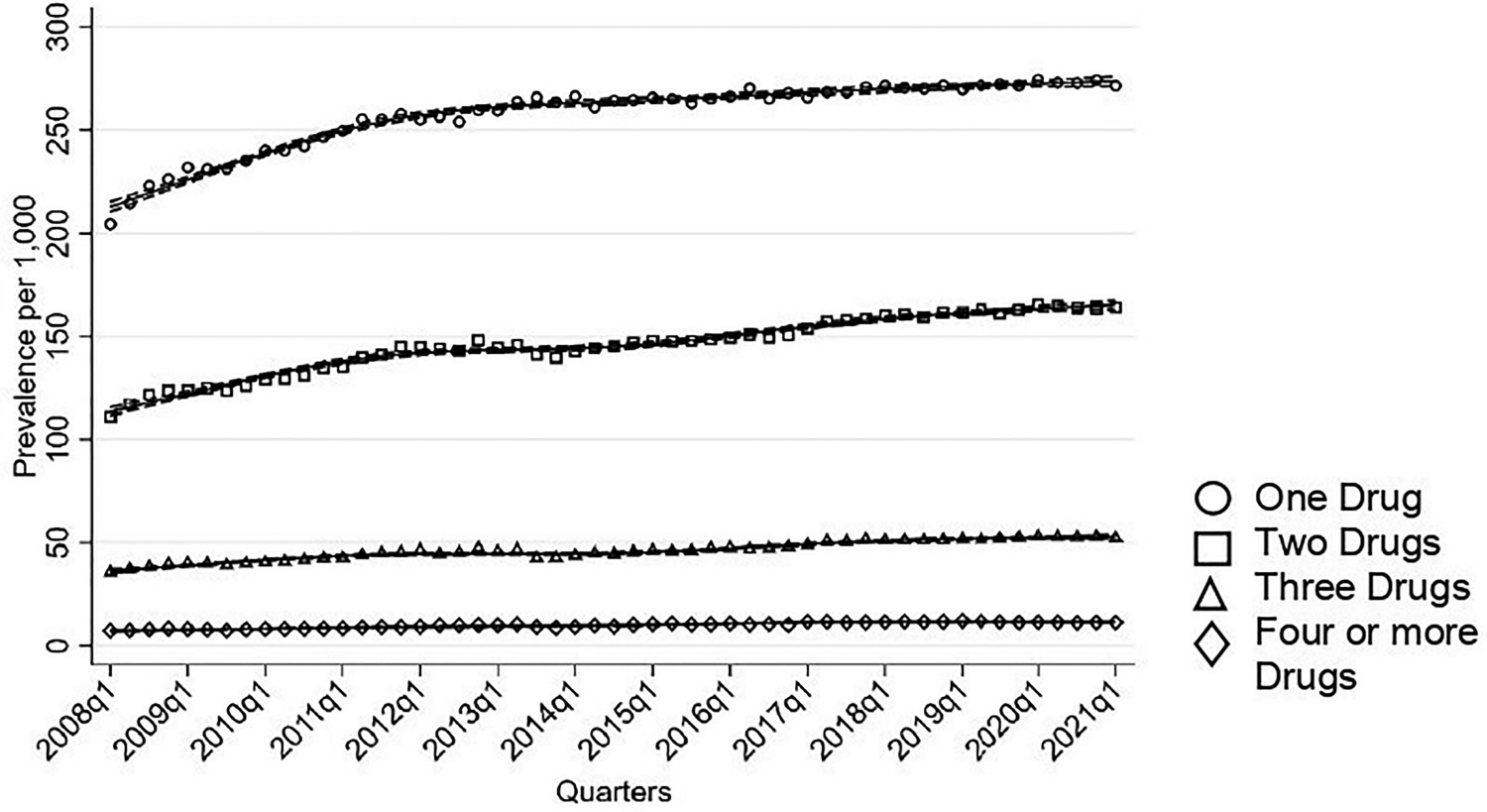
Number of medications used quarterly in total population.

Most individuals were on monotherapy of the drugs investigated in this study, with an increase in treatment rates of monotherapy rising from 204 per 1,000 individuals to 271 per 1,000 (Figure 4). Similarly, treatment rates of combinations of two drugs increased from 111 per 1,000 to 164 per 1,000. An upward trend was seen in individuals using three drugs (36 to 52 per 1,000) and in those on four or more drugs (8 to 11 per 1,000).

**Figure 5.**
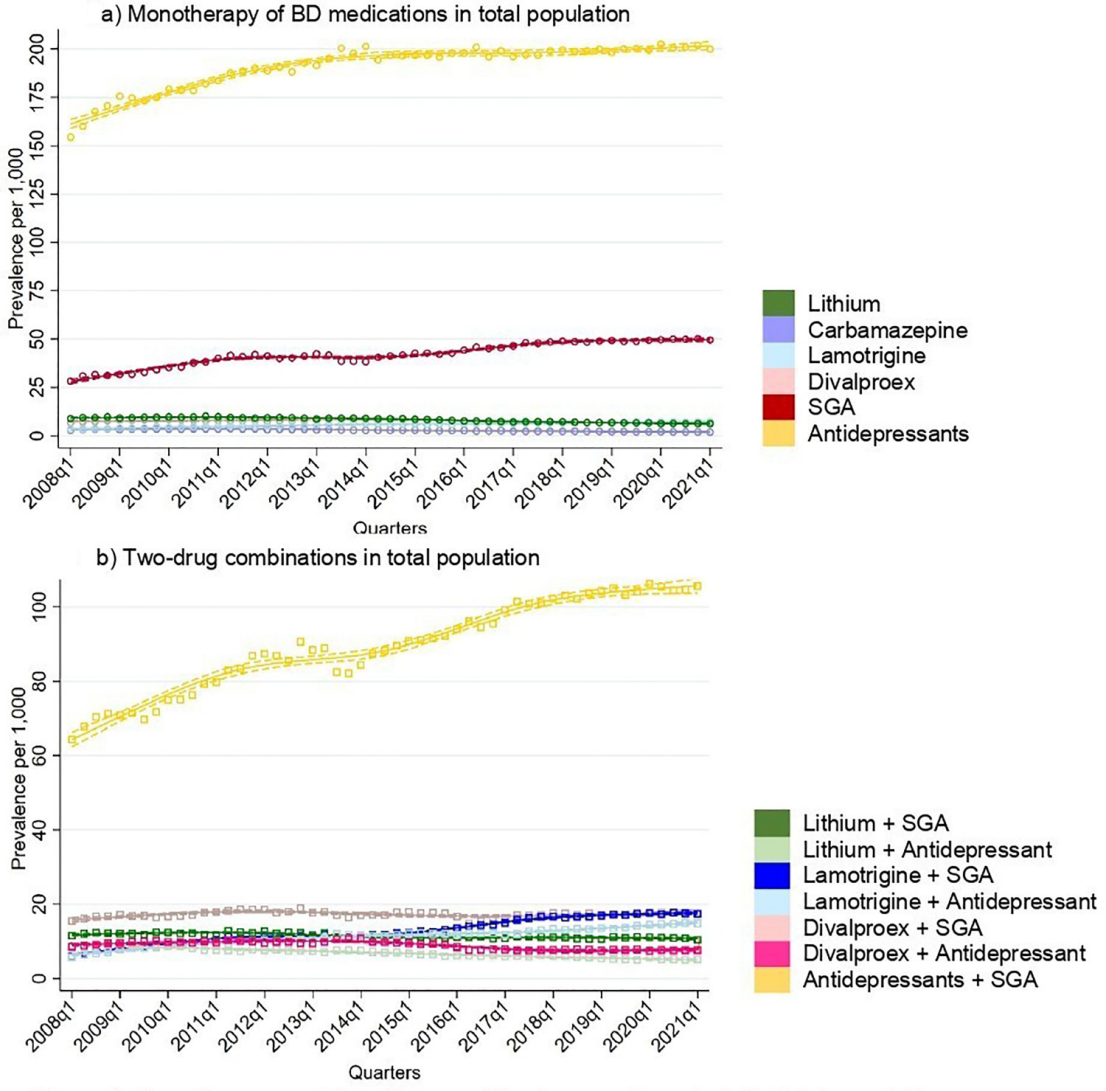
Monotherapy and two-drug combination used quarterly in total population.

Antidepressants were the most common drug class used in monotherapy (Figure 5(a)). Treatment rates of antidepressant as monotherapy increased steadily throughout the study period, rising from 154 per 1,000 to 200 per 1,000. Sensitivity analysis among BD individuals with two or more BD codes showed that treatment rates of antidepressant monotherapy also increased from 125 per 1,000 to 167 per 1,000 by 2013, followed by a decrease in 2021 to 137 per 1,000. Among mood stabilizing medications, treatment rates of monotherapy remained below 10 per 1,000 throughout the observation period.

Antidepressants and SGAs were the most frequently used pairing (Figure 5(b)). The treatment rates of this combination therapy steadily increased, rising from 64 per 1,000 individuals to 106 per 1,000. Treatment rates of lamotrigine combined with either SGAs or antidepressants also showed an increase from 6 per 1,000 to around 18 per 1,000. In contrast, treatment rates of two-drug combinations involving divalproex and lithium with either antidepressants or SGAs remained stable throughout the study period.

## Discussion

This study characterized real-world drug utilization patterns for medications recommended in BD clinical practice guidelines in Alberta, Canada. Our main findings suggest that there is a gap between recommended practice and real world use. First, antidepressant treatment rates, particularly as monotherapy, were higher than expected. Second, SGAs were utilized much more frequently than lithium or divalproex. Notably, lamotrigine treatment rates increased. Finally, we observed a decline in new initiations of all medications, especially after 2020.

Our study reveals high antidepressant use, both as monotherapy and combination therapy, regardless of the number of BD codes. This finding aligns with international trends, where studies report antidepressant use as high as 60% in BD populations.^5,6,8^ However, evidence suggests that antidepressants alone are ineffective for acute and maintenance treatment of depressive episodes, preventing relapses, or reducing suicide risk in BD compared to mood stabilizers.^4,12,13^ Furthermore, antidepressant monotherapy in BD patients may trigger manic episodes and mood dysregulation, particularly in those with mixed features or rapid cycling.^2,13,14^ Our results reflect this concern, as antidepressants were the most commonly prescribed medication class as monotherapy regardless of number of BD codes. Notably, we excluded prescriptions preceding the initial BD diagnosis to minimize potential misclassification of medications used for prior episodes of MDD.

Previous CANMAT guidelines recommended SSRIs or bupropion as first-line adjunctive therapy with mood stabilizers for acute bipolar I depression.^3,15,16^ Other antidepressants, like venlafaxine, monoamine oxidase inhibitors (MAOIs), and TCAs, were considered third-line options in combination with lithium or divalproex.^3,16^ Notably, the 2018 guideline update downgraded the use of SSRIs and bupropion to second-line.^2^ Interestingly, throughout the study period, the most common two-drug combination observed was antidepressants with SGAs. This finding is congruent with the pre-2018 guidelines. After 2018, however, our data suggests a continued high and increasing use of antidepressant-SGA combinations and SSRIs. Furthermore, the rise in other antidepressant use is particularly important given that MAOI and SNRI may have a higher risk (compared to SSRIs) in inducing mood dysregulation in BD patients.^2,14,17^

The observed trend of increased antidepressant treatment rates might be partially attributable to BD II treatment. The CANMAT guidelines from 2007 to 2013 recommended specific antidepressants (bupropion, venlafaxine and fluoxetine) in combination with mood stabilizers or SGAs as a second-line option for acute and maintenance treatment of BDII.^3,15,16^ Recent evidence supporting antidepressant monotherapy for BDII has led to revised CANMAT guidelines recommending venlafaxine, sertraline or bupropion as second-line monotherapy for acute BDII treatment, with venlafaxine additionally recommended for BDII maintenance.^2,18–21^ However, a crucial caveat exists: these recommendations emphasize the use of antidepressants only in patients with “pure depression” and not those with a history of antidepressant-induced hypomania or mixed symptoms. Unfortunately, limitations in administrative data hinder definitive diagnosis between BD I and BD II due to the lack of detailed case ascertainment for BD in general.^22^ The ICD-9 does not include a code for BD II and while ICD-10 does include one (F31.81), it requires a four digit code, which is not generally used in the Alberta data.

Our study indicates that SGAs were the most frequently used BD recommended drugs, with treatment rates towards increasing prevalence. Quetiapine emerged as the dominant SGA, with consistent prescribing patterns throughout the study period. This preference might be attributed to its versatility, being recommended as first-line therapy for both acute and maintenance phases of BD manic and depressive episodes.^2,23–27^ Additionally, quetiapine offers advantages for rapid response, managing co-occurring anxiety, and treating rapid cycling episodes.^28^ Furthermore, its off-label use for comorbid conditions like generalized anxiety disorder and insomnia may contribute to its high overall use.^29^ Importantly, quetiapine is the only SGA with level 1 evidence for both acute and maintenance treatment of BDII.^2,30^ Compared to risperidone and olanzapine, quetiapine may cause fewer metabolic side effects.^29^

The introduction of newer SGAs like aripiprazole (2009) and lurasidone (2012) may have slightly impacted quetiapine's utilization pattern. Treatment rates of aripiprazole steadily increased throughout the study period, potentially due to its first-line recommendation for acute mania since 2009 and the availability of convenient injectable formulations.^2,29,31–33^ Lurasidone, recommended in the 2018 CANMAT guidelines for monotherapy or combination therapy with lithium or divalproex in acute BD depressive episodes, also offers a newer option for this specific indication.^2,3,34,35^ Conversely, the treatment rates of olanzapine and risperidone decreased over the study period. This could be attributed to its association with weight gain and extrapyramidal side effects, despite its availability in injectable forms.^29^

Our study revealed a decline in lithium and carbamazepine treatment rates over time, while divalproex use initially increased followed by a decrease after 2015. Lithium remains a cornerstone mood stabilizer, recommended as first-line therapy for acute and maintenance phases of manic, hypomanic and depressive episodes in both BD I and BD II.^2,3,15,16,36–38^ Divalproex previously held a similar first-line recommendation for both manic and depressive episodes until 2013 CANMAT guidelines downgraded it to second-line for acute depression due to limited evidence.^2,3,39,40^ Despite this downgrade, divalproex continues to have a higher prevalence of both new and prevalent users compared to lithium. This observed preference for divalproex over lithium may be due to lithium's long-term side effects and tolerability issues in some patients.^41–43^

Lamotrigine treatment rates showed a substantial increase, with doubling from 25 to 50 per 1,000 over the study timeframe. Lamotrigine's role in treating both acute and maintenance depressive episodes in BD, coupled with growing evidence for its effectiveness in BD II, likely contributed to its rising popularity.^25,34,44–46^ Furthermore, its relatively safer profile during pregnancy compared to other BD medication, maybe a preferable option, particularly for young women who experience a higher prevalence of bipolar depressive episodes.^2,47^

Lastly, our study suggests that treatment rates of new users of all medications are decreasing. Physicians may be prescribing new patients with BD medications comparatively to a lesser extent. From late 2019 to 2021, new patient diagnosis and treatment and access to medications may have been impacted by COVID-19. Conversely, the expanded availability of treatment options may have empowered individuals to identify and adhere to optimal therapies. Consequently, treatment rates of prevalent user may be increasing as patients maintain longer treatment durations with specific agents, leading to a corresponding decline in new users. Additionally, the early rise in new SGA use may reflect uptake by previously untreated or poorly managed patients, with later declines representing a shift to treating only new-onset.

Our study has several limitations. First, defining a BD cohort solely on at least one BD ICD code for diagnosis may have introduced misclassification bias. The lack of a validated case definition for BD in administrative data potentially may have led to overestimates of drug utilization patterns specifically attributed to BD.^22,48^ To mitigate this concern, we utilized data from multiple databases and conducted a sensitivity analysis focusing on individuals with multiple BD codes. This analysis revealed a consistent pattern of antidepressant use regardless of the number of claims, offering some support for the overall findings. However, future studies may consider evaluating sustained antidepressant use over a defined period to better distinguish between continuous and incident use. Second, while the PIN database captures a high percentage of prescriptions dispensed in community pharmacies, it does not confirm actual medication use by patients.^49^ This limitation may inflate our estimates of both new and prevalent BD drug users. In addition, the rising prevalence of SGA use suggests that the availability of injectable dosage forms might be one of the contributing factors. Further exploration of this possibility is warranted in future research. Finally, the prescribing data analyzed in this study include prescriptions dispensed in Alberta, which may not generalize to other provinces or outside of Canada.

There is a mismatch between clinical guidelines and real-world prescribing practices, possibly due to: (1) guidelines may overlook individual patient complexities, as they are traditionally based on randomized controlled trials, which reflect average outcomes on select populations; (2) guidelines may fail to reflect nuances of clinical care delivered to individual patients, such as comorbidities, patient preferences and past experiences with medications; (3) guideline recommendations may be sound, but knowledge translation may not yet have successfully influenced clinical practice; and (4) pharmaceutical marketing may influence prescribing decisions in ways that deviate from clinical guidance. It is likely that a combination of these factors are relevant. Rapid advances in machine learning and access to large clinical datasets may allow clinical guidance to better reflect individual patient needs alongside RCT evidence. In conclusion, our study suggests a significant shift in BD treatment, with a substantial increase in antidepressant and SGAs treatment rates and a concurrent decrease in lithium and other mood stabilizers over time. The enduring dominance of antidepressant use is especially concerning and warrants further investigation due to potential public health implications, such as increase in relapses, emergency care visits, hospitalizations and mortality. Overall, this study highlights a need for quality improvement initiatives to ensure better alignment of treatment practices with evidence-based guidelines for managing BD.
